# Immobility of the Jaws

**Published:** 1871-01

**Authors:** 


### ARTICLE VIII.

### *Immobility of the Jaws.*

### *Clinic of Prof. Briggs, St. Vincent Hospital.*

JamesD——, aged 21 years, has come to us from Henry County, Tennessee, for relief of an immobility of his jaws, the result of mercury given during an attack of typhoid pneumonia, when he was eight years old. The inflammation resulting from the action of the mercury was very violent, terminating in extensive sloughing, and ulceration of the tissues on the left side of the face, not, however, involving the skin. When the parts healed, the lower jaw was rendered perfectly immovable, and has continued so ever since.

You will observe that the left side of the face is flattened; that it is hard to the touch, but is free from cicatrices. When separating the lips, you see that by his efforts, not the slightest motion of the jaw can be effected; that the upper teeth (more like tusks) are long, and project over the lower; that, on the right side of his lower jaw, two teeth have been extracted, and through this opening he introduces his food and drink.

Upon examination, I find that all the tissues, from the angle of the mouth backwards, are in a state of inodular cicatrization, and firmly adherent to both the upper and lower jaw. I think, but am not perfectly certain, that there is a slight lateral motion of the jaw.

The patient is in a very unpleasant condition, especially for unlocking his jaw; but such operations are not always successful. Sometimes we fail to unloose the jaw at all, but more frequently, after it is separated, it becomes closed again, by the gradual contraction of the inodular cicatrices in the healing process.

I will now divide, within the mouth, all the adhesions between the cheeks and jaws, freely. It may be necessary, at the same time, to divide the masseter muscle and, if I should encounter any osseous plates, I will endeavor to sever them; after which, by means of the screw lever first referred to by Scultetus,and afterwards introduced to the profession by our distinguished countryman, Professor Valentine Mott, I will force the jaws asunder if possible. Either the instrument mentioned, or little wedges of wood, should then be kept in the mouth, day and night, and pieces of slippery-elm bark interposed between the cheeks and the jaws, to prevent them from adhering. I am prepared for obstinate resistance in this case.

(Free incisions were then made through the adhesions, in which was a plate of bone extending from the coronoid process to the upper jaw. This was divided with the bone forceps, and a portion removed, and the screw lever applied; but it was not until the second one had been added, and great force used, that the parts were separated. The jaws were then kept asunder by the instruments, for three weeks, at the end of which time the patient had regained the complete functions of the jaws, and went home, with instructions to keep the wedges in the mouth for a month or two.)—*Nashville Journal of Medicine and Surgery.*

---

### MONTHLY SUMMARY.

